# Monthly Summary

**Published:** 1870-11

**Authors:** 


### MONTHLY SUMMARY.

---

*Carbolic Collodion.*—Dr. J. M. Hirsh of Chicago, has published in a new Journal, *The Arts*, a description of the method of preparing his new mixture for stopping hemorrhage. Taking advantage of the astringent property in carbolic acid, which even in dilute solution will check the flow of blood, he prevents external coagulation—objectionable on account of its liability to break off and allow the flow to commence anew—by adding to the carbolic acid, phenol collodion, which almost instantly forms an artificial covering closely fitting the wound, so that coagulation has to take place within or beneath this coating. Ordinary collodion, which is a solution of gun cotton dissolved in ether, contracts greatly upon the evaporation of the latter, and frequently scales off. To obviate this he uses glycerine, which has the property of rendering the collodion elastic. He finds that carbolate of glycerine is soluble in all proportions in collodion. The highly irritating and poisonous property of carbolic acid suggests that it should be used very sparingly in this compound.—*Medical and Surgical Reporter.*

---

*What Fruit-Syrups are Composed of.*—On examination of a number of fruit-syrups, a chemist in Brussels found them to be grape sugar syrups, colored with analine colors and flavored with a few drops of the common fruit essences. 200 grains of syrup were found to contain 0.05 grains of fuchsine, which is not without danger, on account of the arsenious and arsenic acid frequently contained therein.

Genuine fruit-syrups lose their color by chlorine ; those colored with analine derivatives give at the same time a floculent precipitate, similar to that produced by ammonia in solutions of sesquioxide of iron. Sulphurous acid destroys the color of both ; sulphuric, hydro-chloric, and nitric acid render the color of genuine syrups brighter, and change the artificial ones into yellowish orange. Potassa decolorizes fuchsine syrups, while red fruit-syrups acquire a dirty greenish hue. Carbonate of potash does not change the color of artificial syrups, while the others are colored green. Basic acetate of lead gives with real fruit-syrups a greenish precipitate, with fuchsine syrups a red one.—*Medical and Surgical Reporter.*

---

*Spontaneous Combustion.*—*L' Union Medicale* of the 15th of February, contains an article from the pen of Dr. Bertholle, wherein full details are given of a case of spontaneous combustion. The subject of it was a woman, thirty seven years old, who was addicted to alcoholic drinks. She was found in her room with the viscera and some of the limbs consumed, the hair and clothes having escaped. The very minute description of the state in which the deceased was found, shows that ignition could not have been communicated from without, and, to all appearance, this is an additional case to those already upon record.—*Lancet.*

---

*Influence of Diet on the Composition of Bone.*—Some very interesting experiments have been made by M. Papillon, and communicated to the French Academy. M. Papillon fed pigeons and rats with food containing small quantities of phosphate of strontia, phosphate of magnesia and phosphate of alumina. The substances were given daily in small doses for several months without any visible effect on the health of the animals.

On afterwards making an analysis of the bones of the pigeons, there were in a hundred parts of the ash of the bone:

| | |
|---|---|
| Lime | 46·75 |
| Strontia | 8·45 |
| Phosphoric acid | 41·80 |
| Phosphate of magnesia | 1·80 |
| Residue | 1·10 |
| | 99·90 |

The ash of the bones of the rats furnished in like manner, in Specimen 1:—

| | |
|---|---|
| Lime | 41·10 |
| Alumina | 6·95 |
| Phosphoric acid, &c | 51·95 |
| | 10·00 |

In Specinen 2:—

| | |
|---|---|
| Lime | 46·16 |
| Magnesia | 3·56 |
| Phosphoric acid, &c | 50·29 |
| | 100.00 |

It is very remarkable that strontia, magnesia and alumina can be made to enter into the composition of bone by means of an appropriate diet. A fact of this kind is calculated to raise our hopes in the possibility of modifying the animal body by means of medicines. Possibly ossification of the arteries is capable of being stopped by an appropriate diet.—*Lancet.*

---

*Transformation of the Hydrate of Chloral into Chloroform in the Human Organism.*—M. Personne's communication on Chloral, has been brought before the Academy of Sciences. The results of his experiments point to the transformation of chloral into chloroform within the human organism. His experiments were conducted in two ways. First, chloral was directly mixed with the blood of an ox; next, the substance was given in toxic doses to a dog, which was slain when in a condition of complete anæsthesia. The blood of both animals was examined, and not the slightest trace of chloroform was to be detected by the smell.

But when M. Personne, thinking that the smell of chloroform was necessarily covered by that of the blood, resorted to other means of investigation, he arrived at quite different results. A chemical analysis of the fluid was made, and then it was discovered, by employing the usual means for the detection of chloroform, that a large quantity of chloride of silver could be obtained from the blood which had been directly mixed up with the chloral, and a less quantity from the blood of the dog which had absorbed the substance. As the objection might be made that the chloride of silver does not result trom the transformation of chloral and the consequent introduction of chloroform into the blood, M. Personne at once tells us that he could obtain the chloride of silver only when a small quantity of carbonate of sodium was added to the hydrate of chloral. Thus, he says, the alkali in this experiment has alone transformed chloral into chloroform, as does the alkali of the blood. The same results were obtained on examining the residue found in the stomach of the dog; and M. Personne states that absorption through this channel is extremely slow. Examination of the urine furnished no traces of chloroform—a fact which is in contradiction to what Dr. Bouchet has recently observed.

M. Personne has come to the conclusion "that hydrate of chloral does not pass through the human organism without undergoing a transformation, but that on reaching the blood it is separated into formic acid and chloroform, which latter substance is subsequently converted into chloride of sodium and formiate of soda, the products of its elimination."—*Lancet.*

